# Artificial Intelligence in Gastroenterology: Beyond Diagnostics and Toward Lifestyle and Dietary Interventions For Gastrointestinal Disorders

**DOI:** 10.7759/cureus.100976

**Published:** 2026-01-07

**Authors:** Kunal Ajmera, Om Patel, Nihar Shah

**Affiliations:** 1 Hospital Medicine, Inova Health Systems, Leesburg, USA; 2 Medicine, University of South Florida, Tampa, USA; 3 Medicine, First Physician Group of Sarasota Memorial Hospital, Sarasota, USA; 4 Gastroenterology, Sarasota Memorial Hospital, Sarasota, USA

**Keywords:** ai and machine learning, artificial intelligence and machine learning, artificial intelligence in gastroenterology, artificial intelligence in medicine, digital therapeutics (dtx), food and nutrition, functional gastrointestinal disorder, software-driven digital therapeutics

## Abstract

Gastrointestinal (GI) diseases such as inflammatory bowel disease (IBD), irritable bowel syndrome (IBS), celiac disease, liver cirrhosis, and functional gastroesophageal reflux disorder (GERD) are often associated with a substantial increase in various symptoms, including pain, a reduced quality of life, and the need for medical attention. Dietary and lifestyle changes remain the backbone of treatment for many of these diseases, but they are not always implemented due to obstacles such as low adherence, a lack of customization, inadequate health insurance, and difficulty in accessing expert advice.

Artificial intelligence (AI) has demonstrated significant potential in gastroenterology, particularly in diagnostics such as endoscopy and imaging. However, its therapeutic applications, especially in providing diet and lifestyle support, remain in early stages. To bridge this gap, this narrative review examines the potential of AI to deliver culturally responsive, scalable, and personalized dietary guidance to this patient population. The significance of this approach cannot be overstated, particularly for patients from diverse racial backgrounds and for those who lack access to medical treatment.

AI offers a solution that utilizes natural language processing (NLP), predictive analytics, and real-time patient support, thereby helping to improve adherence, personalize advice, and extend treatment beyond the clinic. One option is to integrate digital medicines, microbiome data, wearables, and AI-driven systems to ensure proactive management of GI problems through continuous monitoring. When AI is developed in an ethical manner that protects data and emphasizes integrity, it can gradually transform the dietary management of GI disorders. AI could help improve GI health by encouraging a more proactive and personalized approach to treatment, increasing patient independence, and reducing unnecessary physician visits.

## Introduction and background

Gastrointestinal (GI) illnesses impose a significant and escalating public health burden, affecting nearly 70 million Americans and incurring annual direct healthcare expenditures of approximately $136 billion, exceeding those associated with heart disease or mental health disorders. Conditions such as irritable bowel syndrome (IBS), gastroesophageal reflux disorder (GERD), inflammatory bowel disease (IBD), gastroparesis, and liver cirrhosis result in substantial healthcare utilization, with millions of emergency department visits, hospital admissions, and procedures annually [1], and significantly affect quality of life and productivity due to persistent symptoms including abdominal discomfort, bloating, diarrhea, and constipation.

Functional gastrointestinal disorders (FGIDs), now referred to as disorders of gut-brain interaction (DGBI) in the Rome IV classification, encompass chronic conditions such as IBS, functional dyspepsia, functional constipation, and functional diarrhea. These disorders result from dysregulation of the gut-brain axis, which affects visceral sensitivity, motility, immune function, and the microbiota [2]. The Rome IV criteria are internationally recognized standardized guidelines for diagnosing disorders of DGBI. DGBIs are characterized by symptoms persisting for a minimum of six months before diagnosis, with specific symptom frequency over the preceding three months, and occurring without structural or biochemical abnormalities that could otherwise explain the symptoms [3].

IBS alone affects approximately 12% of the global population, 4.7% to 5.3% of U.S. adults, accounts for over 40% of GI referrals [4,5], and incurs annual direct medical expenses ranging from $742 to $7,547 [6], contributing to the projected $136 billion economic burden [7]. Gastroparesis affects approximately 1.5% of the U.S. population, and IBD affects over three million Americans, with annual direct costs ranging from $7,824 to $41,829 and national expenditures rising from $6.4 billion in 1996 to $25.4 billion in 2016 [8-11]. Diet plays a major role in many of these GI diseases, with interventions such as low-FODMAP diet (Fermentable Oligosaccharides, Disaccharides, Monosaccharides, and Polyols) for IBS, gluten-free diets for celiac disease, low-sodium diets for cirrhosis, high-fiber diets for constipation, lactose-free diets for intolerance, and lifestyle changes for GERD, all shown to reduce symptoms, healthcare utilization, and disability [12-14]. Therefore, a key avenue for reducing the clinical and financial burden of these highly prevalent illnesses is the integration of evidence-based dietary practices into the management of GI disorders.

Artificial intelligence (AI), which leverages machine learning and deep learning to examine large datasets, identify patterns, and enhance clinical decision-making, is increasingly utilized across medicine, including radiology, cardiology, oncology, and nutrition science, to improve accuracy, efficiency, and personalized care [15-18]. In gastroenterology, the most advanced AI applications are in endoscopy and imaging, where randomized trials (RCTs) and meta-analyses demonstrate higher adenoma and polyp detection rate (PDR), reduced miss rates, enhanced trainee performance, and improved workflow efficiency, with additional applications in capsule endoscopy and the identification of small bowel hemorrhage [19-22].

Despite strong evidence supporting dietary and lifestyle therapies, which often outperform pharmaceutical treatments in gastroenterology, they remain largely underutilized. Dietary interventions face barriers such as limited access to dietitians, low adherence, financial constraints, insufficient personalization, and a lack of real-time guidance, all of which AI could potentially address through scalable, culturally responsive, and patient-centered support [23-26].

This narrative review defines AI-driven nutritional treatments as systems that use machine learning, natural language processing (NLP), or adaptive algorithms to analyze patient-generated or clinical data and continuously refine dietary recommendations over time. This distinction differentiates them from conventional digital health or telehealth platforms that rely on static, rule-based, or clinician-mediated guidance without ongoing model learning or personalization. Building on this framework, the review critically evaluates the role of AI in dietary research within gastroenterology, with particular emphasis on its capacity to provide real-time support for patients managing complex dietary regimens. It synthesizes current evidence on AI-assisted dietary interventions, emphasizing technological approaches, clinical applications, regulatory and ethical considerations, and methodological limitations, while evaluating their potential to improve dietary adherence, symptom management, and overall quality of life.

## Review

Methodology

A structured literature review was performed to evaluate the use of AI in gastroenterology, emphasizing diagnostic and therapeutic applications related to diet and lifestyle interventions. Over 73 peer-reviewed articles retrieved from the PubMed and ScienceDirect databases were analyzed, with findings systematically summarized to facilitate clarity and contextual understanding of current research trends and limitations.

Observations

Current Applications of AI in Gastroenterology

AI has been proven to be highly advantageous in the diagnostic domains in gastroenterology. Multiple RCTs have shown that AI-assisted colonoscopy improves adenoma detection rate (ADR) and polyp detection rate (PDR) compared to traditional procedures. For example, a meta-analysis of 10 RCTs involving 6,629 patients discovered that AI increased ADR by a relative risk (RR) of 1.43, while also improving PDR [19]. Importantly, AI lowers operator-dependent variability, enabling trainees to achieve ADR levels comparable to experts [20].

Beyond colonoscopy, AI models have been designed to assist with histologic scoring in ulcerative colitis, lowering subjectivity, boosting accuracy, and accelerating clinical trials by automating endpoint assessments [26]. AI achieves a gastroenterologist-level accuracy in detecting erosions, ulcers, and bleeding during capsule endoscopy, resulting in increased efficiency by lowering video review times and reducing oversight of small anomalies. Despite the positive results, the use of everyday practice remains restricted due to cost, workflow integration, and regulatory hurdles [21,22].

Diet and Lifestyle as Primary Interventions

Dietary and behavioral interventions continue to be principal or adjunctive treatments for GI diseases. The low-FODMAP diet is one of the most evidence-based treatments for IBS, with many RCTs and network meta-analyses showing substantial reductions in bloating, digestive discomfort, and bowel irregularity [23]. Upon consumption, colonic bacteria rapidly decompose the low-FODMAP diet, which results in gas generation, intestinal distension, and osmotic effects that can contribute to bloating, abdominal discomfort, and alterations in bowel habits. Alternative diets, such as starch- and sucrose-reduced diets (SSRD), have demonstrated efficacy in alleviating similar symptoms while enhancing metabolic indicators, including BMI and fasting glucose levels [24]. Similarly, stringent gluten avoidance is the cornerstone of celiac disease treatment, and it also improves certain IBS patients who have non-celiac gluten sensitivity [25]. Clinical guidelines for GERD emphasize lifestyle modifications, including the avoidance of dietary triggers, late-night eating, and weight reduction, as essential for symptom management [27].

Barriers to Implementation

Despite ample evidence, the implementation of these diets is hindered by difficulties in adherence and access to appropriate healthcare. The patients can experience frustration and discontinue treatment due to complicated dietary restrictions, insufficient access to skilled nutritionists, and a lack of customization. In the clinical trial, the participants' adherence to low FODMAP diets gradually declined, with the estimates suggesting that fewer than 50% of individuals maintained this dietary approach long-term in real-life scenarios [23-25]. Nutritional regimens were initially formulated for Western populations, constraining their cultural relevance. The patients from varied origins can face difficulties in adapting evidence-based diets to locally relevant cuisines.

Another issue is that patients can not get real-time input that connects diet to symptoms, so they must rely on trial and error. Clinicians encounter additional challenges: gastroenterologists often lack sufficient time and resources during regular appointments to provide extensive dietary counselling, and many facilities lack trained dietitians or behavioral specialists [28]. Even when it's available, episodic care makes it difficult to get long-term, context-sensitive guidance. As a result, patients typically rely on unsupervised online resources of varying quality, risking misinformation, nutritional deficiencies, and unnecessary restrictions impacting long-term follow-up strategies and ultimately impeding nutritional control [29]. Patients frequently undertake challenging lifestyle modifications independently, receiving limited assistance from specialists in the absence of integrated support from various disciplines.

AI technologies offer a novel approach to surmount these implementation obstacles. Applications driven by NLP can deliver individualized, literacy-appropriate dietary content, reducing dependence on unreliable web information [30]. Predictive analytics, powered by machine learning, can identify patients at risk of nonadherence or complications, allowing for proactive outreach from the care team [31,32]. AI-powered chatbots and mobile assistants extend physician presence beyond clinic visits, providing reminders, incentives, and real-time support in a scalable and cost-effective fashion [33].

The PROTEIN trial found that AI-driven dietary strategies improved gut microbiome diversity, waist circumference, and food patterns in just six weeks [34]. Similarly, telehealth-based lifestyle coaching following intragastric balloon therapy yielded weight loss results comparable to in-person sessions [35]. During the COVID-19 pandemic, telenutrition and telehealth initiatives improved dietary adherence and health literacy, especially for individuals with limited access to in-person care [36]. Notably, AI systems can help eliminate dietary care disparities by benefiting people who struggle with procuring food, lack knowledge in operating a computer, or are unable to get medical care. AI can offer help in various languages and cultures, and incorporate socioeconomic factors that can impact health into predictive models.

When utilized judiciously, prioritizing the mitigation of bias, safeguarding data privacy, and fostering equitable designs, AI can facilitate access to superior lifestyle support, diminish reliance on erroneous or deceptive information, and bridge the health disparities among vulnerable populations. The ethical literature emphasizes the necessity of training these algorithms on representative datasets to avert bias and ensure equitable benefits for individuals of various races, ethnicities, and socioeconomic statuses [37].

Most of the current AI efforts in gastroenterology focus on diagnostics, including polyp detection, histological grading, and disease classification based on images. The key needs for therapeutic guidance and longitudinal disease management have been conspicuous by their absence. Integration of AI into dietary and lifestyle domains can meet an important, hitherto unmet need. These tools can implement personalized care strategies, offer real-time feedback, and ensure continuous behavioral reinforcement between clinic visits-capabilities that are challenging to accomplish within the limitations of conventional episodic care models. AI-driven platforms can facilitate evidence-based self-management for patients by integrating dietary data with wearables, microbiome sequencing, and physiological monitoring (e.g., pH, manometry). This integration could enable real-time flare prediction and avoidance measures, thereby decreasing reliance on invasive procedures, minimizing dependence on unsupervised information sources, enhancing compliance with dietary and behavioral recommendations, and eventually revolutionizing the management of chronic GI diseases.

Future roadmap

Digital Symptom Logging and AI-Augmented Diet Applications

The development of patient-centered mobile apps incorporating meal logging, symptom tracking, and culturally specific nutritional databases represents a significant opportunity for A in gastroenterology. These systems can provide dynamic, personalized feedback through the correlation of dietary intake patterns with real-time illness progression. Preliminary studies using digital elimination diet platforms enhanced by machine learning algorithms have reported improved compliance and significant symptom reduction in patients with IBS [34,35]. Advances in computer vision technologies, including barcode scanning, image-based portion estimate, and menu recognition, can significantly offset the challenges of human data entry, a primary barrier to long-term patient adoption of digital health apps. These characteristics collectively make AI-enhanced diet apps a viable option for patient-centric, scalable nutritional therapy for FGIDs.

Integration With Wearables, Microbiome, and Predictive Analytics

The subsequent phase will involve integrating wearables, biomarkers, and microbiome profiling with AI-driven dietary systems. Continuous glucose monitors, gut pH sensors, and accelerometers offer physiological correlates of dietary triggers. Machine learning has been utilized to forecast hospitalizations and complications in IBD [38]. Expanding these models to include diet-symptom correlations may facilitate real-time predictions of flare-ups.

FDA-Cleared Digital Therapeutics and Precision Nutrition Ecosystems

In the long term, AI-driven platforms could develop into FDA-approved digital therapeutics (DTx) for conditions such as Functional GI disorders, IBD, functional dyspepsia, and gastroparesis. These tools may be eligible for reimbursement and prescription in standard practice, as there are established precedents for digital health applications in behavioral medicine and diabetes management. The ultimate application of AI may be in augmented daily life, such as adaptive precision nutrition ecosystems, automated cooking tools, and smart eyewear that can help provide dietary advice. These tools have the potential to lower costs substantially by averting the need for invasive procedures and enhancing patient self-management capabilities

Challenges and considerations

Significant hurdles and obstacles for AI applications in dietary management for GI illnesses encompass the necessity for stringent validation, the formulation of regulatory frameworks, ethical dilemmas, and equality issues. Prospective RCTs are essential for evaluating the long-term outcomes, real-world safety, adherence, and clinical value, as evidenced by recent multicenter AI-nutrition trials for IBS

Regulatory monitoring is expected to categorize dietary AI platforms under the FDA's Software as a Medical Device (SaMD) regulation, which demands that the same evidence criteria be met and that algorithmic transparency be assured. Ethical and legal considerations raise significant demands for strong measures to reduce the risks of misinformation, patient harm, ambiguous culpability, and data privacy. This calls for the dissemination of accurate information and the safe handling of personal health data. Diverse and representative datasets should be used to ensure precise AI performance in providing nutritional advice that is appropriate for people of different cultural, ethnic, and dietary backgrounds. This can help close the gap in healthcare and promote more patient-centered care.

## Conclusions

AI significantly influences gastroenterology, although strong evidence is primarily concentrated in diagnostic applications, particularly in colonoscopy and histologic grading. The therapeutic domain, especially applications involving dietary and lifestyle modification of GI conditions, remains underdeveloped. AI offers a new approach to long-standing challenges related to adherence, personalization, and real-time support for patients with IBS, IBD, functional dyspepsia, and related functional disorders, as diet is closely linked to symptom management. Preliminary applications suggest that AI-enhanced nutrition platforms, digital symptom-monitoring tools, and telehealth coaching can improve adherence, enhance patient engagement, and achieve outcomes comparable to standard in-person management. Advances in wearables, microbiome sequencing, and predictive analytics are likely to enable real-time flare forecasting and provide highly personalized dietary counseling. Ultimately, such systems may evolve into FDA-approved digital therapies that are reimbursable and integrated into standard clinical care, extending evidence-based nutritional support beyond the clinic.

To fully realize this potential, large-scale clinical validation will be required, along with clear governmental regulations and oversight, to ensure outcome accuracy, patient safety, and equitable benefits for all stakeholders involved. Addressing bias and ensuring cultural adaptability will be critical to making dietary AI guidance effective and accessible across diverse populations. When thoughtfully developed, AI has the near-term potential to significantly enhance gastroenterological dietary management by empowering patients, reducing healthcare costs, and minimizing inequities in care. AI holds substantial promise for gastroenterology, as it can extend care beyond the hospital setting into the daily lives of patients. From detection to prevention, and from intermittent consultations to continuous support, AI could transform gastrointestinal treatment into a truly personalized, proactive, and patient-centered paradigm.

## References

[1] Peery AF, Murphy CC, Anderson C (2025). Burden and cost of gastrointestinal, liver, and pancreatic diseases in the United States: update 2024. Gastroenterology.

[2] Drossman DA, Hasler WL (2016). Rome IV-functional GI disorders: disorders of gut-brain interaction. Gastroenterology.

[3] Ray G, Ghoshal UC (2024). Epidemiology of disorders of the gut-brain interaction: an appraisal of the Rome IV criteria and beyond. Gut Liver.

[4] Lacy BE, Patel NK (2017). Rome criteria and a diagnostic approach to irritable bowel syndrome. J Clin Med.

[5] Almario CV, Sharabi E, Chey WD, Lauzon M, Higgins CS, Spiegel BM (2023). Prevalence and burden of illness of Rome IV irritable bowel syndrome in the United States: results from a nationwide cross-sectional study. Gastroenterology.

[6] Canavan C, West J, Card T (2014). Review article: the economic impact of the irritable bowel syndrome. Aliment Pharmacol Ther.

[7] Peery AF, Crockett SD, Murphy CC (2022). Burden and cost of gastrointestinal, liver, and pancreatic diseases in the United States: update 2021. Gastroenterology.

[8] Camilleri M, Parkman HP, Shafi MA, Abell TL, Gerson L (2013). Clinical guideline: management of gastroparesis. Am J Gastroenterol.

[9] Jung HK, Choung RS, Locke GR 3rd (2009). The incidence, prevalence, and outcomes of patients with gastroparesis in Olmsted County, Minnesota, from 1996 to 2006. Gastroenterology.

[10] Kahn-Boesel O, Cautha S, Ufere NN, Ananthakrishnan AN, Kochar B (2023). A narrative review of financial burden, distress, and toxicity of inflammatory bowel diseases in the United States. Am J Gastroenterol.

[11] Singh S, Qian AS, Nguyen NH (2022). Trends in U.S. health care spending on inflammatory bowel diseases, 1996-2016. Inflamm Bowel Dis.

[12] Camilleri M, Chedid V, Ford AC (2018). Gastroparesis. Nat Rev Dis Primers.

[13] Parrish CR (2015). Nutritional considerations in the patient with gastroparesis. Gastroenterol Clin North Am.

[14] Homko CJ, Duffy F, Friedenberg FK, Boden G, Parkman HP (2015). Effect of dietary fat and food consistency on gastroparesis symptoms in patients with gastroparesis. Neurogastroenterol Motil.

[15] Esteva A, Robicquet A, Ramsundar B (2019). A guide to deep learning in healthcare. Nat Med.

[16] Hosny A, Parmar C, Quackenbush J, Schwartz LH, Aerts HJ (2018). Artificial intelligence in radiology. Nat Rev Cancer.

[17] Guha A, Shah V, Nahle T (2025). Artificial intelligence applications in cardio-oncology: a comprehensive review. Curr Cardiol Rep.

[18] Sosa-Holwerda A, Park OH, Albracht-Schulte K, Niraula S, Thompson L, Oldewage-Theron W (2024). The role of artificial intelligence in nutrition research: a scoping review. Nutrients.

[19] Huang D, Shen J, Hong J (2022). Effect of artificial intelligence-aided colonoscopy for adenoma and polyp detection: a meta-analysis of randomized clinical trials. Int J Colorectal Dis.

[20] Bernhofer S, Prosenz J, Venturi D, Maieron A (2025). The impact of artificial intelligence on the adenoma detection rate: comparison between experienced, intermediate and trainee endoscopists' adenoma detection rate. Wien Klin Wochenschr.

[21] Ding Z, Shi H, Zhang H (2019). Gastroenterologist-level identification of small-bowel diseases and normal variants by capsule endoscopy using a deep-learning model. Gastroenterology.

[22] Mascarenhas M, Ribeiro T, Afonso J (2022). Deep learning and colon capsule endoscopy: automatic detection of blood and colonic mucosal lesions using a convolutional neural network. Endosc Int Open.

[23] Cuffe MS, Staudacher HM, Aziz I (2025). Efficacy of dietary interventions in irritable bowel syndrome: a systematic review and network meta-analysis. Lancet Gastroenterol Hepatol.

[24] Roth B, Nseir M, Jeppsson H, D'Amato M, Sundquist K, Ohlsson B (2024). A starch- and sucrose-reduced diet has similar efficiency as low FODMAP in IBS-a randomized non-inferiority study. Nutrients.

[25] Nybacka S, Törnblom H, Josefsson A (2024). A low FODMAP diet plus traditional dietary advice versus a low-carbohydrate diet versus pharmacological treatment in irritable bowel syndrome (CARIBS): a single-centre, single-blind, randomised controlled trial. Lancet Gastroenterol Hepatol.

[26] Peyrin-Biroulet L, Adsul S, Stancati A, Dehmeshki J, Kubassova O (2024). An artificial intelligence-driven scoring system to measure histological disease activity in ulcerative colitis. United European Gastroenterol J.

[27] Ajmera K, Thaimuriyil N, Shah N (2022). Recent advances in the endoscopic management of gastro-esophageal reflux disorder: a review of literature. Cureus.

[28] Scarlata K, Eswaran S, Baker JR, Chey WD (2022). Utilization of dietitians in the management of irritable bowel syndrome by members of the American College of Gastroenterology. Am J Gastroenterol.

[29] Ribaudi E, Amato S, Becherucci G (2025). Addressing nutritional knowledge gaps in inflammatory bowel disease: a scoping review. Nutrients.

[30] Redd D, Workman TE, Shao Y (2023). Patient dietary supplements use: do results from natural language processing of clinical notes agree with survey data?. Med Sci (Basel).

[31] Selya A, Anshutz D, Griese E, Weber TL, Hsu B, Ward C (2021). Predicting unplanned medical visits among patients with diabetes: translation from machine learning to clinical implementation. BMC Med Inform Decis Mak.

[32] Mirzadeh SI, Arefeen A, Ardo J (2022). Use of machine learning to predict medication adherence in individuals at risk for atherosclerotic cardiovascular disease. Smart Health (Amst).

[33] Zhang J, Oh YJ, Lange P, Yu Z, Fukuoka Y (2020). Artificial intelligence chatbot behavior change model for designing artificial intelligence chatbots to promote physical activity and a healthy diet: viewpoint. J Med Internet Res.

[34] Rouskas K, Guela M, Pantoura M (2025). The influence of an AI-driven personalized nutrition program on the human gut microbiome and its health implications. Nutrients.

[35] Vargas EJ, Bazerbachi F, Storm AC (2019). Effectiveness of online aftercare programs following intragastric balloon placement for obesity is similar to traditional follow-up: a large propensity matched US multicenter study. Obes Surg.

[36] Kaufman-Shriqui V, Sherf-Dagan S, Boaz M, Birk R (2021). Virtual nutrition consultation: what can we learn from the COVID-19 pandemic?. Public Health Nutr.

[37] Abrahams M, Raimundo M (2025). Perspective on the ethics of AI at the intersection of nutrition and behaviour change. Front Aging.

[38] Gan RW, Sun D, Tatro AR, Cohen-Mekelburg S, Wiitala WL, Zhu J, Waljee AK (2021). Replicating prediction algorithms for hospitalization and corticosteroid use in patients with inflammatory bowel disease. PLoS One.

